# Effect of Molar Distalization on Condyle-Glenoid Fossa Relationship

**DOI:** 10.1155/2023/5549951

**Published:** 2023-06-27

**Authors:** Dani Abo Samra, Rania Hadad, Omar Hamadah

**Affiliations:** ^1^Department of Orthodontics and Dentofacial Orthopaedics, Faculty of Dental Medicine, Damascus University, Damascus, Syria; ^2^Department of Oral Medicine, Higher Institute for Laser Research and Applications, Faculty of Dental Medicine, Damascus University, Damascus, Syria

## Abstract

**Objective:**

It is essential to be aware of the potential effects of orthodontic treatment on tissues and anatomical structures associated with the masticatory system, especially the temporomandibular joint (TMJ). Little information is available about the consequences of molar distalization on the TMJ. Therefore, this study is aimed at investigating the changes of the condyle-fossa relationship after molar distalization using the distal jet appliance.

**Materials and Methods:**

The sample consisted of twenty-five patients (mean age 20.4 ± 2.6) who underwent molar distalization by the distal jet appliance. CBCT scans were taken before (T0) and after (T1) the completion of the molar distalization. Joint spaces (anterior, superior, and posterior) and cephalometric vertical angles (SN.GOME and Björk sum) were measured and compared at T0 and T1.

**Results:**

Superior and posterior joint spaces increased significantly after molar distalization (PS 0.29 mm, *P* < 0.001, SS 0.06 mm, *P* < 0.5). Vertical cephalometric angles also increased after molar distalization by the distal jet appliance (SN.GOME 0.92°, Björk 1.11°).

**Conclusion:**

There was a statistically significant increase in the superior and posterior joint spaces after molar distalization. However, this increase may not be of clinical importance. The vertical dimension has also increased.

## 1. Introduction

Molar distalization is a nonextraction treatment option to gain space in the maxillary arch or to correct the class II molar relationship [1].

Molar distalization causes not only dental changes but also skeletal changes. Clockwise rotation of the mandibular plane and the increase in mandibular plane angle may be produced when molars are distalized into the wedge of the occlusion [2, 3]. These modifications may directly or indirectly affect the temporomandibular joint (TMJ) [4].

Currie et al. have studied the changes in mandibular condylar pathways after maxillary molar distalization with a pendulum and concluded that molar distalization leads to significant changes in axiography values, and these changes seem to correspond to the alterations of the mandibular movements [5].

Cone beam computed tomography (CBCT) provides high-resolution multiplanar and cost- and dose-effective imaging for the evaluation of a variety of TMJ without superimposition [6–8]. CBCT seems to be superior to conventional radiographical examinations and MRI in assessing the morphology of the osseous joint components, bone morphology, joint spaces, and dynamic function in all three dimensions [6, 7].

Since accomplishing static and dynamic occlusion is a very important goal in orthodontic treatment for the maintenance of healthy teeth, jaws, and surrounding hard and soft tissue structures [9], it is necessary to understand the changes that may occur in the TMJ during orthodontic treatment. Many researchers have investigated the effects of rapid maxillary expansion (RME) on the TMJ [4, 10, 11], changes in the TMJ disc-condyle-fossa relationship following functional treatment of skeletal class II [12–14], and the effect of the protraction facemask on this joint [15].

On the other hand, there is no study yet that has investigated the influences of molar distalization on the TMJ.

The ideal position of the condyle in the glenoid fossa and how various nonskeletal orthodontic treatments affect this relationship are still ambiguous with very few studies in the literature. A posterior rotation of the mandible can be expected by molar distalization which may also affect the position of the condyle in the fossa. Thus, this study is aimed at evaluating the changes in the TMJ after the treatment with the distal jet appliance (DJ) using CBCT.

## 2. Materials and Methods

This study was approved by the institutional review board and ethical review committee of Damascus University (Damascus, Syria; institutional review board no. 3945).

The CBCT images of 25 patients (mean age 20.4 ± 2.6 years; females 23 and males 2) were retrospectively selected from the archive of molar distalization research. The inclusion criteria were as follows:

- Class II malocclusion, division 1 or 2
- Horizontal or average growth patterns
- Harmony of the facial profile
- Absence of transversal discrepancies
- Minimal or no crowding in the mandibular arch

Patients with the absence of dental units, previous extraction, craniofacial deformities involving condyles and/or mandible, history of rheumatic diseases, signs or symptoms of TMJ disorder, history of orofacial tumors, and those with previous orthodontic treatment were excluded from the study.

All procedures were explained to the patients and the informed consent was signed. Molar distalization was achieved using DJ (American Orthodontics, Sheboygan, Wisconsin) for each subject. The clinical procedures were as follows [16]: tooth separation, bands fitting on the maxillary first premolars and maxillary first molars, correct and complete alginate impression and dental casts, space maintenance during the construction of DJ in the laboratory, fitting of the DJ and checking the force generated by the coil spring (240 grams per side), luting using glass ionomer cement (GIC), and coil spring activation every 4 weeks until achieving class I molar relationship (with overcorrection).

CBCT images were taken before treatment (T0) and at the end of molar distalization and removing the DJ (T1). All CBCT images were carried out at the same device VATECH (Pax-i3D Green, Seoul, Korea), performed under constant settings, and were of 0.25 mm^3^ voxel size with the field of view (FOV) 15 × 15 and 9-second scan time. The patients' heads were oriented by locating the Frankfurt plane parallel to the floor while they remained in maximum dental intercuspation.

The same operator (DAS) made all measurements, who was blinded to the patient's name and the time point of the image (T0 or T1). The images were saved in digital imaging and communications in medicine (DICOM) format and then were viewed and measured with the software Ez3Dplus (Seoul, Korea).

**Figure 1:**
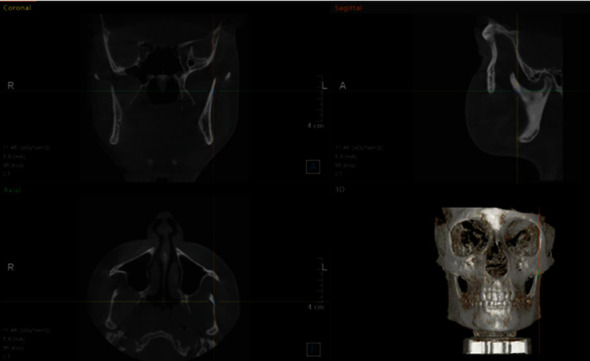
Standardization CBCT image for condyle-glenoid fossa measurements.

**Figure 2:**
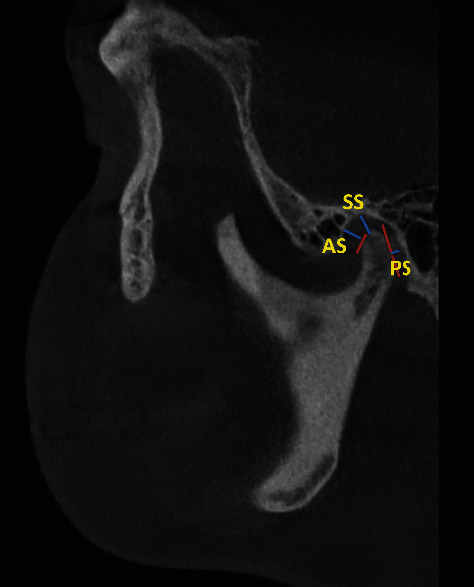
Landmarks and linear measurements. Lines (the red lines) tangent to the most prominent anterior and posterior points of the condyle. The superior joint space (SS), the anterior joint space (AS), and the posterior joint space (PS).

CBCT images were standardized for measurements of the space between the condyle and glenoid fossa as follows [17]: The yellow axis was positioned tangent through the pterygoid vertical, the orange axis was located along the center of the sigmoid notch (on the axial section), and the green axis passed tangent to the sigmoid notch (on the sagittal section) (Figure 1). The condyle-fossa relationship was evaluated by measuring anterior, superior, and posterior joint spaces [17, 18] (Table 1 and Figure 2).

To evaluate the vertical dimension changes, SN.GOME and Björk sum were measured at T0 and T1.

All measurements for 15 randomly chosen CBCT scans were repeated by the same examiner (DAS) after one month to assess the repeatability of the measurements.

## 3. Statistical Analysis

Intraclass correlation coefficients (ICC) were calculated to assess the repeatability of the measurements. To determine whether the changes in the joint spaces between the right and left sides were significant, an independent Student's *t*-test was applied, and the results of this test have shown that there were no significant differences between the two sides (Table 2). Therefore, the right and left joint spaces were grouped together, and a total of 50 TMJs comprised the sample. Descriptive statistics (mean, standard deviation) were calculated. A paired *t*-test was carried out to evaluate whether the changes in the joint spaces (AS, SS, and PS), and the vertical cephalometric angles before and after treatment were significant.

## 4. Results

The ICC for each measurement was ≥0.99 demonstrating excellent examiner reliability (Table 3).

Vertical cephalometric angles increased after molar distalization and SN.GOME increased 0.92°, while Björk's sum was 1.11° bigger than values before treatment (Table 4).

Measurements of the joint spaces (AS, SS, and PS) showed that SS exhibited the highest value both before and after molar distalization (3.63 mm and 3.68 mm, respectively), followed by PS (2.64 mm and 2.93 mm, respectively) and AS (2.48 mm and 2.50 mm, respectively) (Table 5).

There was a statistically significant increase in the superior and posterior joint spaces after molar distalization (PS 0.29 mm, *P* < 0.001, SS 0.06 mm, *P* < 0.5), whereas the increase in the anterior joint space was insignificant (Table 5).

## 5. Discussion

Recently, several modern methods have been developed to distalize the maxillary dental arch and treat class II malocclusion without extraction, such as class II Carriere Motion appliance [19], modified C-palatal plates [20], and temporary skeletal anchorage devices [21]. Lately, TMJ analysis and the effect of different orthodontic treatments on joint spaces have gained increased interest from researchers. Torres et al. concluded that rapid maxillary expansion in growing patients could cause a positional change of the condyle within the glenoid fossa but has no effects on the position or shape of the articular disc [4]. On the other hand, Nindra et al. did not find any significant change in the condyle-fossa relationship following treatment with the Herbst appliance which induced growth at the condylar head and anterior remodeling of the glenoid fossa [17]. Similarly, Cacho et al. reported that after the treatment of class II with activator, no differences in any direction in condyle position were detected [22]. To the best of our knowledge, this study is the first 3-dimensional research that is aimed at evaluating the changes in the joint spaces after molar distalization by DJ.

Since the CBCT is a reliable method to assess the joint spaces and condylar volume in different planes [23, 24], it was used widely in the research to evaluate the outcome of many orthodontic treatments that may affect the TMJ [4, 10, 11, 22]. To avoid unnecessary radiation exposure, the CBCT data were retrospectively collected from the archive of molar distalization research. Moreover, all CBCT images included in the current study were taken by the same device (VATECH) with only 9 seconds of exposure time which minimizes greatly radiation exposure. The panoramic and cephalometric images were obtained from the CBCT scans without the need for any additional radiographic images to perform the orthodontic diagnosis and measurements.

The main advantage of the DJ appliance is that it allows almost translatory molar distalization [25, 26]. Some studies found no significant vertical changes during distalization by DJ [25, 27], whereas other studies confirmed that this kind of treatment could cause clockwise rotation of the mandible plane [2, 3]. According to Reis et al. [3], DJ stimulated an increase (0.7 ± 2.0°) in the mandibular plane angle which was explained by the extrusion, distalization mechanics, and tipping of the maxillary second molars. Because of these conflicting results and the potential importance of these changes on the TMJ, this research included also an evaluation of the vertical dimension variables. The findings of the current study confirmed a minimal but statistically significant increase in SN.GOME angle and Björk sum. This increase in the vertical height and the clockwise rotation of the mandible made studying the effects of molar distalization on the TMJ of high importance.

**Figure 3:**
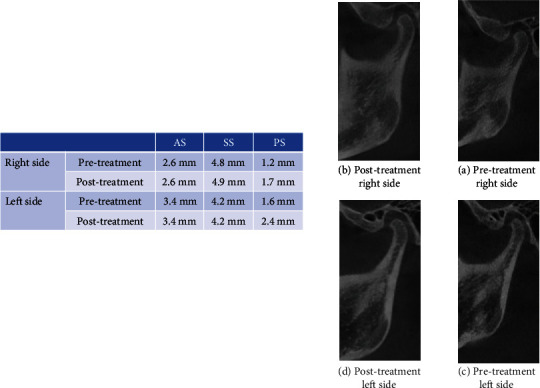
Case 1 (female, 21 y). CBCT evaluation of condyle-glenoid fossa relationship changes after molar distalization with distal jet. (a, c) Pretreatment and (b, d) posttreatment (right and left, respectively).

**Figure 4:**
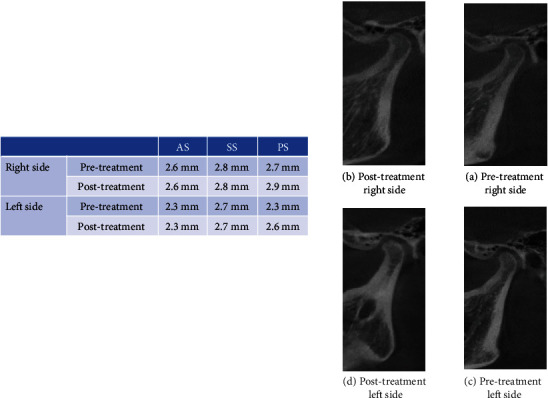
Case 2 (female, 18 y). CBCT evaluation of condyle-glenoid fossa relationship changes after molar distalization with distal jet. (a, c) Pretreatment and (b, d) posttreatment (right and left, respectively).

In this study, the measurements of the joint spaces (AS, SS, and PS) showed that the superior and posterior joint spaces have significantly increased after molar distalization (Figures 3 and 4). This result can be explained by the clockwise rotation of the mandible that is caused by molar distalization. This alteration in the relationship between the condyle and the fossa may explain the change of the mandibular condylar pathways after maxillary molar distalization that was documented by Currie et al. [5].

Although these changes in the joint spaces were statistically significant, they were minimal (SS 0.06 mm, PS 0.29 mm) and did not seem to be clinically appreciable. However, it must be taken into account that all the patients included in this study were with horizontal or average growth patterns and have relatively little change in vertical cephalometric angles (SN.GOME increased 0.92°) observed. Molar distalization in patients with a vertical growth pattern may cause larger clockwise rotation and more changes in the joint spaces. However, such a vertical growth pattern is a contraindication to molar distalization. On the other hand, one limitation of the current study is that no control groups were comprised, yet avoiding untreated patients from radiation exposure was an ethical concern.

Nevertheless, the strength of this study stems from the fact that it was the first CBCT research about the changes in the TMJ following molar distalization which could open new avenues for dental research about this important topic, especially that many modern orthodontic treatments are directed toward treatment without tooth extraction.

## 6. Conclusion

There is a minimal but statistically significant increase in the superior and posterior joint spaces after molar distalization in patients with average or horizontal growth patterns. These changes did not seem to be clinically significant which may confirm the safety of this procedure on the TMJ. Distal jet appliance promotes an increase in the vertical cephalometric angles (SN.GOME, Björk sum).

## Figures and Tables

**Table 1:** Linear measurements of the space between the condyle and glenoid fossa.

Measurements	Abbreviations	Definition
Anterior joint space	AS	The vertical distance from the anterior Tangent point to the glenoid fossa
Superior joint space	SS	The distance from the most superior condyle point to the highest point on the glenoid fossa
Posterior joint space	PS	The vertical distance from the posterior Tangent point to the glenoid fossa

**Table 2:** Independent Student's *t*-test comparing the changes of right and left joint spaces.

Joint space	Side	Mean	Std. D.	Mean difference	*P* value
AS	Right	0.01	0.11	0.00	1.000
	Left	0.01	0.13		
SS	Right	0.03	0.17	-0.05	0.324
	Left	0.08	0.18		
PS	Right	0.27	0.31	-0.04	0.629
	Left	0.31	0.33		

**Table 3:** Intraclass correlation coefficients (ICC) for all repeated measurements.

Variable	ICC value
SN.GOME	1.000
B	1.000
Björk sum	0.999
Right AS	0.996
Right SS	0.999
Right PS	0.998
Left AS	0.996
Left SS	0.998
Left PS	0.998

**Table 4:** Paired *t*-test comparing vertical cephalometric angles before and after molar distalization.

Angle	T0		T1		Mean difference	*P* value
	Mean	SD	Mean	SD		
SN.GOME	28.66	5.43	29.58	5.35	0.92	<0.001^∗∗∗^
Björk sum	388.61	5.39	389.72	5.35	1.11	<0.001^∗∗∗^

^∗∗∗^ *P* < .0001.

**Table 5:** Paired *t*-test comparing joint spaces before and after molar distalization.

Joint space	*N*	T0		T1		Mean difference	*P* value
		Mean	SD	Mean	SD		
AS	50	2.48	0.60	2.50	0.57	0.01	0.472
SS	50	3.63	0.85	3.68	0.85	0.06	0.024^∗^
PS	50	2.64	0.67	2.93	0.74	0.29	<0.001^∗∗∗^

^∗^ *P* < .05 and ^∗∗∗^*P* < .0001.

## Data Availability

The datasets used and/or analyzed during the current study are available from the corresponding author on reasonable request.
